# Initial pupil status is a strong predictor for in-hospital mortality after aneurysmal subarachnoid hemorrhage

**DOI:** 10.1038/s41598-020-61513-1

**Published:** 2020-03-16

**Authors:** Marius M. Mader, Andras Piffko, Nora F. Dengler, Franz L. Ricklefs, Lasse Dührsen, Nils O. Schmidt, Jan Regelsberger, Manfred Westphal, Stefan Wolf, Patrick Czorlich

**Affiliations:** 1 Department of Neurosurgery, University Medical Center Hamburg-Eppendorf, Martinistraße 52, 20246 Hamburg, Germany; 2 Department of Neurosurgery, Charité-Universitätsmedizin Berlin, Charitéplatz 1, 10117 Berlin, Germany; 3 Institute for Stem Cell Biology and Regenerative Medicine, Stanford University School of Medicine, 265 Campus Drive, Stanford, CA 94305 USA; 4 Department of Neurosurgery, University Medical Center Regensburg, Franz-Josef-Strauss-Allee 11, 93053 Regensburg, Germany

**Keywords:** Medical research, Stroke, Stroke, Eye manifestations

## Abstract

Prognosis of patients with high-grade aneurysmal subarachnoid hemorrhage (aSAH) is only insufficiently displayed by current standard prognostic scores. This study aims to evaluate the role of pupil status for mortality prediction and provide improved prognostic models. Anonymized data of 477 aSAH patients admitted to our medical center from November 2010 to August 2018 were retrospectively analyzed. Identification of variables independently predicting in-hospital mortality was performed by multivariable logistic regression analysis. Final regression models included Hunt & Hess scale (H&H), pupil status and age or in a simplified variation only H&H and pupil status, leading to the design of novel H&H-Pupil-Age score (HHPA) and simplified H&H-Pupil score (sHHP), respectively. In an external validation cohort of 402 patients, areas under the receiver operating characteristic curves (AUROC) of HHPA (0.841) and sHHP (0.821) were significantly higher than areas of H&H (0.794; p < 0.001) or World Federation of Neurosurgical Societies (WFNS) scale (0.775; p < 0.01). Accordingly, including information about pupil status improves the predictive performance of prognostic scores for in-hospital mortality in patients with aSAH. HHPA and sHHP allow simple, early and detailed prognosis assessment while predictive performance remained strong in an external validation cohort suggesting adequate generalizability and low interrater variability.

## Introduction

Aneurysmal subarachnoid hemorrhage (aSAH) represents a severe disease associated with high mortality and morbidity^1^. Prognostic scores are essential tools to provide the treatment team with a first overall expression of the clinical status of the patient and assist with treatment guidance. Well-established scores are the Hunt & Hess scale (H&H) and the World Federation of Neurosurgical Societies (WFNS) scale mainly considering extent of neurological symptoms and level of consciousness^2,3^. A frequently used radiographic score is the Fisher grading system correlating the extent of blood in computed tomography with the risk for vasospasm^4^. Given the importance of patient classification according to injury severity and prognosis for clinical routine and study inclusion, evaluation and optimization of prognostic scores for aSAH is an ongoing and desirable endeavor. Therefore, prognostic scores of other acute cerebral diseases like intracerebral hemorrhage (ICH) – e.g. the modified Graeb score or the SAH score (HAIR) as an adaption of ICH score – have been investigated regarding their utility in aSAH^5,6^. Equally, trauma scores, e.g. in the simplest form the Glasgow Coma Scale (GCS), have been evaluated in aSAH as well^7,8^. Moreover, established aSAH scores have been adapted like the WFNS herniation scale (hWFNS) considering signs of brainstem herniation^9^. Similarly, the importance of pupil status for prognostic stratification has been outlined for traumatic brain injury (TBI) by the two recently established grading systems GCS-Pupils score (GCS-P) and the Eppendorf-Cologne Scale (ECS)^10,11^. A dilated and fixed pupil can be a sign of increased intracranial pressure that led to uncal herniation and compression of the ipsilateral oculomotor nerve where it crosses the sphenoid bone^12^. Further deterioration leads to subsequent change in reactivity and size of also the opposite pupil^13^. The aim of this retrospective study was to investigate the independent influence of pupil status, evaluate the prognostic value of scores considering pupil status like GCS-P and ECS in patients with aSAH and propose novel aSAH scores for in-hospital mortality prediction.

## Methods

The study was performed in accordance with international ethical standards and institutional guidelines/regulations. Anonymized data of all patients with diagnosis of aSAH admitted to our tertiary medical center from November 2010 to August 2018 were retrospectively analyzed with approval from the local ethics committee (Ethik-Kommission der Ärztekammer Hamburg, WF-069/18). The study is exempt from the need for informed consent under local law (Hamburger Krankenhausgesetz §12). Ethics of the external validation cohort was previously reported^14^.

Aneurysmal nature of aSAH was verified by cerebral digital subtraction angiography, computed tomography angiography or magnetic resonance angiography. All patients were admitted to specialized neurocritical care units of our center. Aneurysm treatment modalities were microsurgical or endovascular depending on an interdisciplinary consensus between the departments of neurosurgery and neuroradiology.

Prognostic data including GCS, H&H, WFNS and pupil status were based on clinical status at first encounter with a physician. In Germany, this is usually already the case in prehospital emergency care. Accordingly, if available, data were based on ambulance documentation. Otherwise, emergency department documentation served as data source. Inhomogeneous data regarding GCS, H&H and WFNS mainly due to divergent time points of grading were reviewed. Patients with incoherent data demonstrating GCS > 13 and H&H > 3 as well as patients with H&H > 3 and WFNS < 3 were excluded from analysis. Moreover, patients with missing or unclear documentation of GCS, GCS motor score or pupil status were excluded from analysis as well.

Further data included basic demographic as well as clinical information such as gender, age, Fisher scale, presence of intraventricular hemorrhage (IVH), rebleed, delayed cerebral ischemia (DCI) and cardiopulmonary resuscitation (CPR). DCI was defined as previously described by Vergouwen *et al*.^15^. Primary outcome parameter was in-hospital mortality.

Statistical analysis was performed using IBM® SPSS® Statistics Version 24.0 (IBM Corporation, Armonk, NY, USA). Descriptive data are presented as mean ± standard deviation (SD) or median and interquartile range (IQR) as applicable. Univariate analysis was performed using Pearson Chi-Square test or Fisher’s Exact Test as appropriate. A level of statistical significance of p < 0.05 was applied. Identification of variables independently predicting in-hospital mortality was performed by multivariable logistic regression analysis. For this, linear variable age was categorized to <60, 60–79 and >80 years of age as previously described for the HAIR score^6^. GCS was categorized as proposed by Takagi *et al*. (GCS 15, GCS 11–14, GCS 8–10, GCS 4–7, GCS 3)^8^. Odds ratios (OR), 95% confidence intervals (CI) and p values were reported. For the development of novel scoring systems, assignment of points was based on regression coefficients of the final logistic regression models. As a measure of internal validation of the model and in order to compare the predictive performance between novel and established aSAH scores, receiver operating characteristic (ROC) curves and corresponding areas under the curve (AUC) were calculated. Differences between areas under the ROC of novel scores and well-established H&H and WFNS were statistically tested by DeLong’s test using R 3.5.2 (R Foundation, Vienna, Austria) and pROC package 1.13.0^16,17^.

Findings were externally validated in a tertiary medical center aneurysmal aSAH cohort of 402 patients which has been described previously^14^.

## Results

### Characteristics of the study cohort and univariate analysis

In total, 513 patients with an aSAH were considered in this retrospective analysis. Thirty-six patients were excluded due to incongruent or ambiguous data. Thus, statistical analysis was based on a collective of 477 patients. The majority of patients was female (67.1%). Mean age was 55.2 ± 13.4 years. A normal pupil status was present in 89.3% whereas unilaterally and bilaterally dilated pupils were present in 6.9% and 3.8% of patients, respectively. Median GCS was 15.0 (IQR 7.0–15.0). Aneurysm occlusion was performed endovascularly in 310 (65.0%) and microsurgically in 132 (27.7%) patients. In 7.3% of patients, no aneurysm treatment was performed due to poor clinical condition. Acute hydrocephalus requiring external ventricular or lumbar drain placement was present in 303 (64.1%) patients. DCI occurred in 184 (38.8%) of cases. We observed in-hospital mortality in 97 (20.5%) patients. A diagnosis of brain death was determined in 41 patients (43.2%). A therapy was discontinued due to other medical reasons i.e. sepsis or lung failure in 14 patients (14.7%). A withdrawal of care in accordance to advance directives or presumed patient will was present in 40 patients (42.1%). The mean time from bleed to brain death diagnosis was 5.3 ± 4.7 days. The mean interval from bleed to withdrawal of care was 8.9 ± 7.3 days. Table 1 provides an overview on clinical features including established aSAH scores and associated mortality in the presented cohort. Age, GCS, pupil status, acute hydrocephalus, rebleed, IVH, CPR, H&H, WFNS, Fisher scale, hWFNS and HAIR exhibited a statistically significant association with mortality in univariate analysis (p < 0.05).

**Table 1:** Univariate analysis.

Variable	Category	Total	Mortality Rate	p value
Sex	female	320 (67.1%)	20.1%	0.808
	male	157 (32.9%)	21.3%	
Age groups [years]	<60	316 (66.2%)	15.0%	<0.001
	60–79	140 (29.4%)	28.6%	
	>80	21 (4.4%)	47.6%	
GCS categorized	15	247 (51.9%)	6.1%	<0.001
	11–14	73 (15.3%)	13.7%	
	8–10	27 (5.7%)	33.3%	
	4–7	57 (12.0%)	36.8%	
	3	72 (15.1%)	59.2%	
Motor score	6	306 (64.2%)	6.9%	<0.001
	5	29 (6.1%)	34.5%	
	4	25 (5.2%)	28.0%	
	3	25 (5.2%)	32.0%	
	2	14 (2.9%)	42.9%	
	1	76 (15.9%)	57.3%	
Pupil status	normal	426 (89.3%)	13.9%	<0.001
	anisocoric	33 (6.9%)	66.7%	
	bilaterally dilated	18 (3.8%)	88.9%	
Acute hydrocephalus	present	303 (64.1%)	24.3%	0.009
	absent	170 (35.9%)	14.1%	
Rebleed	present	69 (14.5%)	34.3%	0.005
	absent	407 (85.5%)	18.2%	
IVH	present	283 (71.1%)	29.6%	<0.001
	absent	115 (28.9%)	9.6%	
CPR	present	27 (5.7%)	73.1%	<0.001
	absent	448 (94.3%)	17.4%	
DCI	present	184 (38.8%)	22.8%	0.350
	absent	290 (61.2%)	19.0%	
H&H	1	105 (22.0%)	4.8%	<0.001
	2	145 (30.4%)	6.9%	
	3	79 (16.6%)	14.1%	
	4	50 (10.5%)	32.0%	
	5	98 (20.5%)	56.7%	
WFNS	1	244 (53.3%)	5.8%	<0.001
	2	32 (7.0%)	12.5%	
	3	12 (2.6%)	18.2%	
	4	56 (12.2%)	28.6%	
	5	114 (24.9%)	50.4%	
Fisher	1	19 (4.1%)	16.7%	<0.001
	2	47 (10.0%)	6.4%	
	3	86 (18.3%)	7.0%	
	4	317 (67.6%)	27.0%	
hWFNS	1	244 (51.2%)	5.8%	<0.001
	2	32 (6.7%)	12.5%	
	3	12 (2.5%)	18.2%	
	4	112 (23.5%)	29.7%	
	5	77 (16.1%)	57.1%	
HAIR	0	64 (16.1%)	1.6%	<0.001
	1	124 (31.2%)	8.1%	
	2	79 (19.9%)	15.6%	
	3	22 (5.5%)	45.5%	
	4	14 (3.5%)	57.1%	
	5	46 (11.6%)	52.2%	
	6	39 (9.8%)	56.4%	
	7	8 (2.0%)	75.0%	
	8	1 (0.3%)	100.0%	

### Logistic regression models and scoring system development

We used multivariable logistic regression models to investigate the independent influence of significant variables of the univariate analysis. H&H, GCS and motor score have not been included simultaneously due to overlapping features and consequent correlation. We preferred GCS over motor score, since mortality increased in a more stepwise fashion with GCS. Combination of pupil status with either GCS or H&H demonstrated a Nagelkerke’s R^2^ of 0.361 and 0.374, respectively. Adding variable age to these models yielded further improvement of the model (GCS-Pupil-Age model (GCS-PA): 0.402; Hunt & Hess-Pupil-Age model (HHPA): 0.415) and variables maintained a significant influence on mortality. OR, CI and p-values are depicted in Table 2.

**Table 2:** Multivariable logistic regression analysis.

Model	Nagelkerke’s R^2^	Variable		Regression coefficient	OR	CI - lower	CI - upper	p value
GCS-P_SAH_	0.361	GCS	15 (reference)					<0.001
			11–14	0.89	2.43	1.04	5.68	0.04
			8–10	1.83	6.25	2.33	16.75	<0.001
			4–7	1.89	6.62	3.00	14.57	<0.001
			3	2.26	9.61	4.27	21.64	<0.001
		Pupil status	normal (reference)					<0.001
			anisocoric	1.33	3.77	1.61	8.84	0.002
			bilaterally dilated	2.57	13.12	2.70	63.80	0.001
		Constant		−2.73	0.07			<0.001
GCS-PA	0.402	GCS	15 (reference)					<0.001
			11–14	0.88	2.40	1.01	5.71	0.048
			8–10	1.60	4.97	1.78	13.88	0.002
			4–7	1.89	6.62	2.95	14.89	<0.001
			3	2.22	9.20	3.98	21.27	<0.001
		Pupil status	normal (reference)					<0.001
			anisocoric	1.38	3.98	1.66	9.54	0.002
			bilaterally dilated	2.79	16.21	3.27	80.40	<0.001
		Age	<60 (reference)					<0.001
			60–79	0.66	1.93	1.07	3.46	0.028
			>80	2.03	7.62	2.73	21.24	<0.001
		Constant			0.05			<0.001
H&H-P	0.374	Hunt & Hess	1 (reference)					<0.001
			2	0.40	1.49	0.49	4.50	0.477
			3	1.17	3.21	1.06	9.66	0.038
			4	2.02	7.53	2.52	22.53	<0.001
			5	2.59	13.33	4.72	37.64	<0.001
		Pupil status	normal (reference)					<0.001
			anisocoric	1.26	3.51	1.50	8.22	0.004
			bilaterally dilated	2.52	12.48	2.65	58.76	0.001
		Constant			0.05			<0.001
HHPA	0.415	Hunt & Hess	1 (reference)					<0.001
			2	0.49	1.63	0.53	5.01	0.392
			3	1.18	3.25	1.06	10.00	0.039
			4	2.02	7.56	2.46	23.22	<0.001
			5	2.59	13.27	4.60	38.31	<0.001
		Pupil status	normal (reference)					<0.001
			anisocoric	1.31	3.72	1.55	8.90	0.003
			bilaterally dilated	2.74	15.44	3.22	74.04	<0.001
		Age	<60 (reference)					<0.001
			60–79	0.71	2.02	1.12	3.65	0.019
			>80	2.01	7.48	2.66	21.09	<0.001
		Constant			0.03			<0.001

If added separately to either the GCS-PA or HHPA model, the variables IVH, CPR and acute hydrocephalus showed no significant independent influence (p > 0.05). Whereas the incidence of rebleeding demonstrated a significant independent influence when added to the GCS-PA or HHPA model (OR 2.88 (CI 1.43–5.79), p = 0.003 and OR 2.88 (CI 1.43–5.79), p = 0.003, respectively). However, we did not include the variable to the final model since we only considered factors already available upon admission.

Based on the final regression models of GCS-PA and HHPA, we developed clinical scoring systems by assigning point values to the categories of the variables according to the regression coefficients. A prognosis score is obtained by addition of point values. The grading system of the newly developed GCS-Pupil-Age score (GCS-PA) and H&H-Pupil-Age score (HHPA) are shown in Table 3. Based on the regression coefficients without age, we also developed a simplified Hunt & Hess-Pupil score (sHHP) as a direct easy-to-use grading scale for clinical routine (Table 3). sHHP demonstrated a Nagelkerke’s R^2^ of 0.378 which was slightly superior to simple addition of pupil value to H&H similar to the GCS-P score of Brennan *et al*. (0.376). Other scale constructs like addition of pupil score only to H&H 4 and/or 5 as well as scoring pupil status separately from H&H yielded inferior grading models (Nagelkerke’s R^2^: 0.372, 0.368 and 0.373, respectively).

**Table 3:** Grading systems of novel scores.

Score	Variable		Point value
**GCS-PA**	GCS	15	0
		11–14	2
		8–10	3
		4–7	4
		3	5
	Pupil status	normal	0
		anisocoric	3
		bilaterally dilated	6
	Age [years]	<60	0
		60–79	1
		>80	4
	**MAXIMUM**		**15**
**HHPA**	Hunt & Hess	1	0
		2	1
		3	2
		4	4
		5	5
	Pupil status	normal	0
		anisocoric	3
		bilaterally dilated	5
	Age [years]	<60	0
		60–79	1
		>80	4
	**MAXIMUM**		**14**
**sHHP**	Hunt&Hess	Pupil status	Score
	1	normal	1
	2	normal	2
	3	normal	3
	4	normal	4
	5	normal	5
	4	anisocoric	6
	5	anisocoric	7
	4	bilaterally dilated	8
	5	bilaterally dilated	9

### Evaluation of the predictive performance of aSAH scores

**Figure 1:**
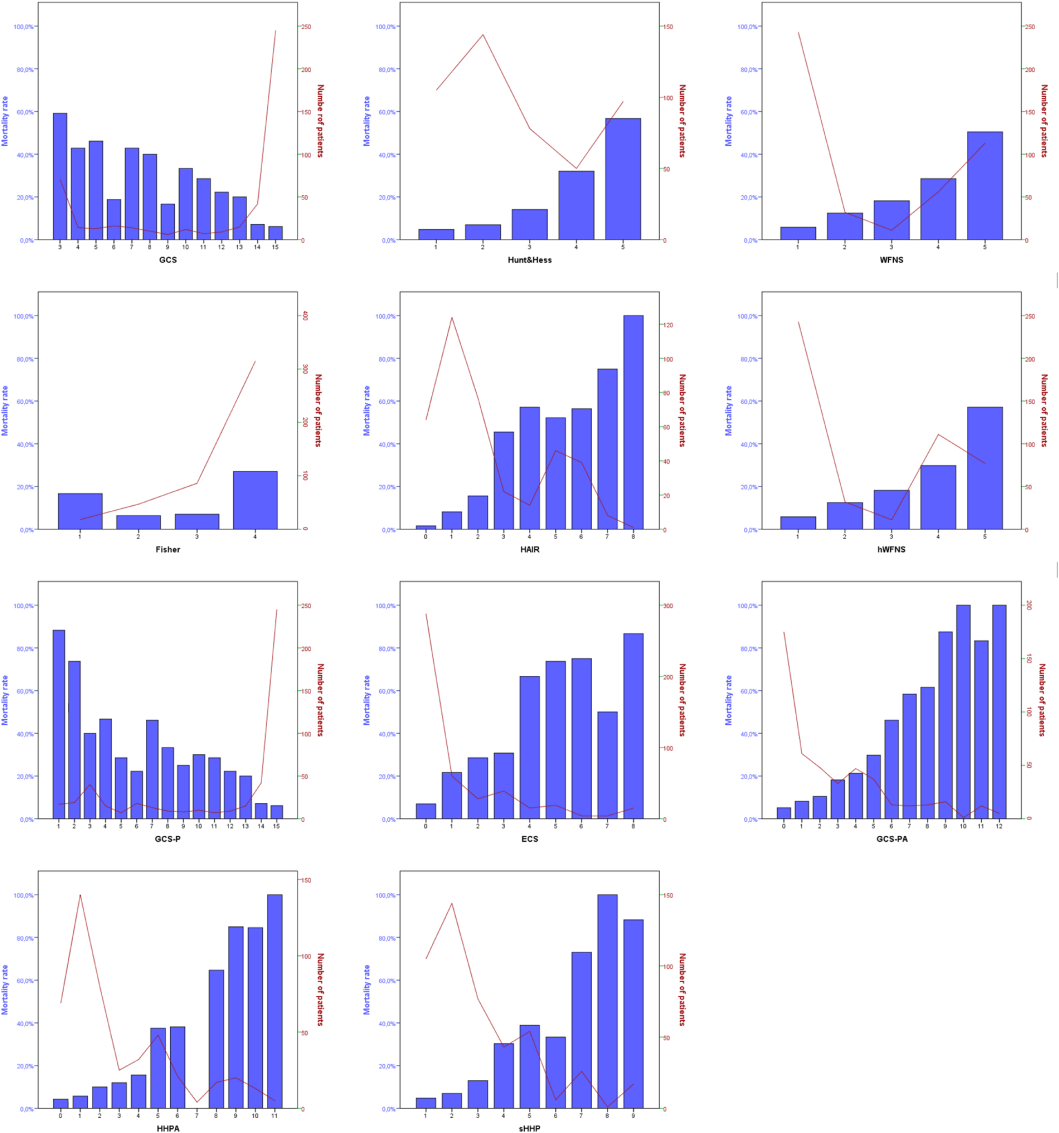
Bar charts (left y axis: blue color) of different established and novel subarachnoid hemorrhage prognosis scores representing in-hospital mortality rate in the derivation cohort are shown. Integrated line plots (right y axis: red color) demonstrate the absolute number of patients allocated to the particular grade.

Figure 1 provides an overview about patient distribution and mortality rates of different established and novel scores. A H&H grade V and WFNS grade V resulted in mortality rates of 56.7% and 50.4%, respectively, while the mortality rate of a hWFNS grade V increased to 57.1%. GCS of 3 exhibited a mortality rate of 59.2% whereas GCS-P of 3, 2 and 1 showed mortality rates of 40.0%, 73.7% and 88.2%, respectively. ECS demonstrated a major increase in mortality from a score of 4 (66.7%). HAIR showed major rises in mortality from 2 (15.6%) to 3 (45.5%) and from 6 (56.4%) to 7 (75.0%), while a score of 8 was represented by a single deceased patient. GCS-PA, HHPA and sHHP exhibited increasing mortality rates in conjunction with higher scores. Increments appeared most steady with GCS-PA until a score of 9. A score of 9 and above represented a mortality rate between 83.3% and 100%. HHPA and sHHP displayed rather stepwise rises. Major mortality increases of HHPA were apparent between 4 (15.6%) and 5 (37.5%) as well as between 8 (64.7%) and 9 (85.0%). As an exception, a score of 7 was only given to few patients and no death event was observed in our cohort. With regards to sHHP, major increases in mortality rate were present between 3 (13.0%) and 4 (30.2%) as well as between 6 (33.3%) and 7 (73.1%).

Supplementary Fig. 1 demonstrates the reclassification from established scores to the grades of HHPA or sHHP. Whereas there was less variation in low grade SAH, a H&H or WFNS grade of 4 or 5 was assigned to various grades of the pupil related scale. For example, starting from a H&H grade 5, resulting HHPA grades were 5, 6, 8, 9, 10 and 11 with 33.7%, 17.3%, 11.2%, 20.4%, 12.2% and 5.1%, respectively. Moreover, this distribution differed between survived and deceased patients. Pupil-related scores showed a distribution of higher grades in the mortality group compared to the survival group within the same H&H or WFNS grade.

**Figure 2:**
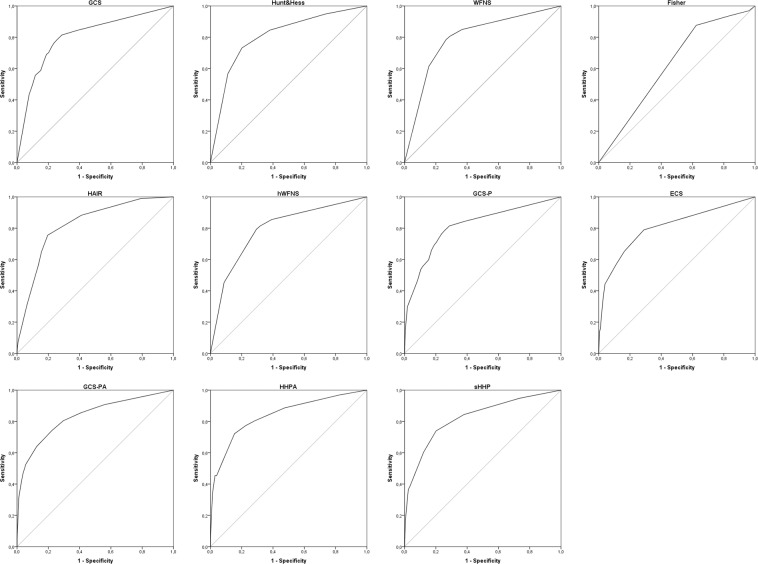
Receiver operating curves of different established and novel subarachnoid hemorrhage prognosis scores in the derivation cohort are demonstrated. Predicted outcome parameter is in-hospital mortality. A reference line is depicted in grey.

Figure 2 and Table 4 show areas under the ROC (AUROC) of the different prognosis scores for mortality. AUROC of HHPA (0.839) and sHHP (0.824) were significantly higher than the areas of H&H (0.808; p = 0.003 and p = 0.003, respectively) or WFNS (0.793; p = 0.001 and p = 0.004, respectively). AUROC of HAIR (0.823; vs H&H: p = 0.129; vs WFNS: p = 0.055), hWFNS (0.794; vs H&H: p = 0.248; vs WFNS: p = 0.574) and ECS (0.805; vs H&H: p = 0.896; vs WFNS: p = 0.197) showed no statistically significant difference to AUROC of H&H or WFNS. AUROC of GCS-P (0.813) and GCS-PA (0.832) were significantly larger than WFNS AUROC (p = 0.037 and p = 0.008, respectively) but differed not significantly from H&H AUROC (p = 0.67 and p = 0.105, respectively).

**Table 4:** Areas under the receiver operating characteristic curves for in-hospital mortality prediction in the derivation cohort.

Score	Area	CI - lower	CI - upper
GCS	0.803	0.751	0.855
H&H	0.808	0.757	0.858
WFNS	0.793	0.741	0.846
Fisher	0.624	0.565	0.682
HAIR	0.823	0.776	0.869
hWFNS	0.794	0.742	0.845
GCS-P	0.813	0.760	0.865
ECS	0.805	0.749	0.860
GCS-PA	0.832	0.782	0.883
HHPA	0.839	0.790	0.888
sHHP	0.824	0.773	0.874

The influence of pupil-related scores on the interval from bleed to withdrawal of care was tested in a linear regression model and showed adjusted R² values of 0.078 (p = 0.045), 0.026 (p = 0.160) and 0.043 (p = 0.105) for HHPA, sHHP and GCS-P, respectively (Supplementary Fig. 2).

**Figure 3:**
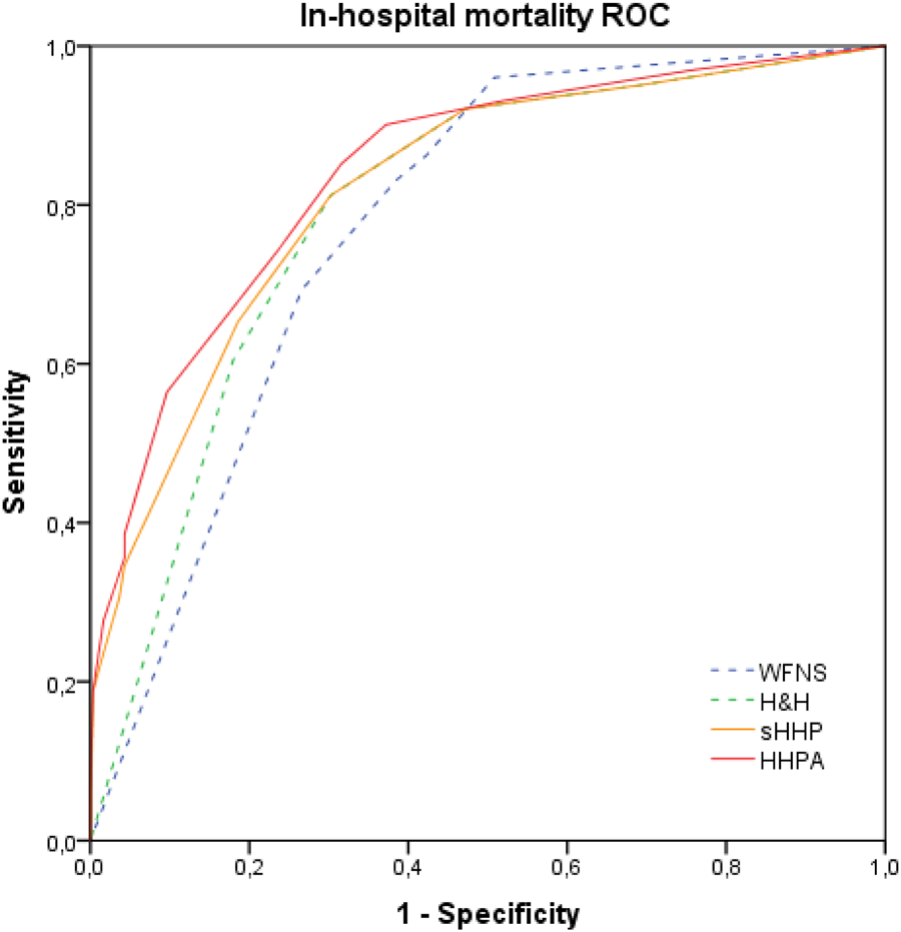
The receiver operating curve for in-hospital mortality of WFNS, H&H, sHHP and HHPA in the validation cohort is shown.

Predictive performance of HHPA and sHHP were confirmed by external validation in a second cohort of aSAH patients (Fig. 3 and Table 5). The external cohort has previously been described^14^. After exclusion of cases due to missing values, 402 patients were valid for analysis. AUROC of HHPA (0.841) and sHHP (0.821) were significantly higher than AUROC of H&H (0.794; p < 0.001 and p < 0.001, respectively) and WFNS (0.775; p < 0.001 and p = 0.003, respectively).

**Table 5:** Areas under the receiver operating characteristic curves for in-hospital mortality prediction in the external validation cohort.

Score	Area	CI - lower	CI - upper
WFNS	0.775	0.728	0.822
HH	0.794	0.746	0.843
HHPA	0.841	0.797	0.886
sHHP	0.821	0.773	0.868

## Discussion

The need for further elaboration of prognostic aSAH scores results from the fact that most severely affected patients are only insufficiently displayed by current ‘gold standard’ scores like H&H or WFNS which are most widely used. This is evident from relatively low mortality rates for H&H grade IV and V aSAH patients as e.g. reported by Le Roux *et al*. (25.0% and 60.2% mortality after 6 months, respectively) which is similar to the presented study cohort (32.0% and 56.7% in-hospital mortality, respectively)^18^. These poor-grade patients represent an inhomogeneous cohort and it is comprehensible that mortality rate is reported even lower for actively treated subgroups showing a mortality rate of 22.9% for H&H grade V patients after three months^19^. Therefore, further prognostic separation of poor-grade patients already upon admission appears desirable for clinical management and study enrollment. This requires the inclusion of a variable highly predictive for mortality which is already available in an early stage of disease.

Considering prognostic models in TBI, level of consciousness and pupil status represent major predictive features as recently demonstrated by the GCS-P as a novel score^10,20^. This has also been modeled as ECS using pupil reactivity, size and modified motor score^11,21^. However, in aSAH, pupil status in particular has rarely been integrated in prognostic models and grading systems even though it represents an easy assessable feature and pupil reactivity has already been associated with long-term outcome in poor-grade aSAH patients^22^. Moreover, pupil dilation at admission was a predictor of in-hospital mortality in an univariate analysis of the Swiss Study on Aneurysmal Subarachnoid Hemorrhage database^23^.

An integration of consciousness and brainstem reflexes in general has been performed for aSAH patients with the FOUR score and the hWFNS^9,24,25^. Hereby, hWFNS grade V demonstrated a higher 6 months mortality rate than WFNS grade V (77.9% vs 68.3%) which is congruent to our findings even though in-hospital mortality rate remained rather moderate (57.1% vs 50.4%) and hWFNS AUROC did not differ significantly from WFNS or H&H AUROC^9^.

Other recently published aSAH prognostic models which were based on large-scale aSAH-cohorts include the SAFIRE scale, FRESH score and different scores based on the SAHIT cohort^26–28^. The SAFIRE model predicted poor functional outcome with an AUROC of 0.73 in the validation cohort and was based on aneurysm size, age, Fisher grade and WFNS^26^. The FRESH score considered variables H&H, age, APACHE-II Physiologic score and aneurysmal rebleed within 48 hours^27^. External validation was performed for functional outcome measured by Modified Rankin Scale after 3 months and yielded an AUROC of 0.769. With regards to mortality prediction after 2–12 months, the SAHIT models demonstrated AUROC of 0.76–0.78 and included age, hypertension and WFNS as well as in expanded variations also neuroimaging information and treatment modalities^28^.

Another recently published aSAH prediction model for in-hospital mortality is the HAIR score as an adaption of the ICH score considering H&H, age, IVH and rebleed^6^. Evaluation of HAIR in the presented study cohort demonstrated a higher AUROC than H&H and WFNS but the difference reached no significance. In our analysis, rebleed event was assessed for the whole length of stay instead of just for the first 24 hours as applied in the original publication. As a result, considering patients with late onset rebleed, who are likely to represent patients with withdrawal of treatment due to a palliative setting, possibly led to an improved AUROC of HAIR. In the literature, in-hospital mortality was also predicted by the SAH score with the variables GCS, age and medical comorbidities demonstrating an AUROC of 0.821 although in an unvalidated setting^29^.

Since GCS-P and ECS demonstrated good predictive performance in TBI, we evaluated the prognostic utility of these novel trauma scores for aSAH^10,11^. In comparison to GCS, GCS-P allowed further subclassification of GCS grade 3 patients. Interestingly, GCS-P grade 3 was associated with a lower mortality than GCS-P grade 4. This paradoxical finding was not apparent for GCS-P but for GCS in the original TBI publication^10^. Overall, GCS-P yielded a larger AUROC than GCS and notably also a significantly larger AUROC than WFNS while a major advantage is certainly the intuitive and easy-to-use format. ECS, however, demonstrated no significant advantage over established scores and showed mainly one major increment from grade 3 to grade 4 instead of a steady increase.

In order to develop an optimized prediction model for mortality, we performed a logistic regression model and included either categorized GCS or H&H as variables for consciousness. GCS was categorized as previously recommended^8^. We chose GCS over motor score due to a steadier mortality increase and H&H over WFNS due to an a priori superior predictive performance. Combination with pupil status and age led to an improved model. Including rebleed as a variable would have further improved the model. However, it was not considered since we focused on variables already available upon admission. Moreover, rebleed might be difficult to detect objectively and may therefore not be an optimal variable for a prognostic score. The final model utilizing H&H as a more aSAH specific parameter yielded a better mortality prediction than the GCS-based variant. This was also reflected by the AUROC analysis with the HHPA score achieving a significantly larger AUC than established standard scores H&H and WFNS. The superior predictive performance was confirmed in the external validation (0.841). HHPA might therefore be a feasible complementary grading system for early mortality prediction in aSAH patients. The role of HHPA should not be a replacement of other established scores but aiding to provide a summarized picture of a patient’s disease severity and prognosis already upon admission. This might be particularly interesting for screening assessments for clinical aSAH studies.

In terms of daily clinical routine, a prognostic score should be easy to use and simple while avoiding too many variables and more complex arithmetic. Therefore, we aimed to develop also a simpler score focusing on just two variables in a scale-like configuration. The sHHP performed best in comparison to other tested simplified models combining H&H and pupil status. Despite its more rudimentary design, it achieved a significantly larger AUROC than H&H and WFNS in both derivation and validation cohort, thus might represent a convenient alternative to HHPA for clinical practice.

There are several limitations of this study and the developed scores. First, the retrospective nature of data inherits the risk of incomplete or incorrect information, which may have affected the presented results. Patient evaluation and documentation of data but also retrospective interpretation of data was subject to inter-rater variability. Second, retrospective risk modelling might represent a ‘self-fulfilling prophecy’ since patients with a poor clinical grade are more likely to be subjected to withdrawal of care or therapy limitations like DNR order or abstaining from aneurysm occlusion^30^. Particularly age should be considered cautiously in this context. The linear regression model exploring the influence of pupil-related scores on the interval from bleed to withdrawal of care could be interpreted in this way (Supplementary Fig. 2). A significant effect was seen for HHPA with shorter time periods to a withdrawal decision associated with higher scores even though the effect size appeared rather minor in the presented study cohort. Last, the role of pupil status and derived models of this study were evaluated and fitted for in-hospital mortality as an objective and early outcome measure similar to other scores like the HAIR score or SAH score of Naval *et al*.^6,29^. Further evaluation and validation or possibly adaption of HHPA and sHHP regarding predictive performance for other timepoints or outcome measures appears to be of interest.

The strength of HHPA and sHHP clearly lies within their objectivity and simplicity. The models are based on only three and two items, respectively. The scores refrain from more complex or ambiguous variables like imaging data or information of the medical history. All variables are available directly upon admission and no events of the further clinical course are required for consideration. This allows for a prognosis assessment as early as possible. Scoring is based on very basic clinical assessment and should minimize interrater bias. This is reflected in the robust validation of the scores in an independent external cohort.

## Conclusions

Including information about pupil status can improve predictive performance of prognostic scores for in-hospital mortality in patients with aSAH. Particularly more detailed description of poor-grade patients seems to be facilitated. For this purpose, GCS-P, a recently developed TBI score, represents an easy-to-use option. Predictive performance could be further improved by combining H&H and pupil status while maintaining a simple scale-like format as proposed with the newly developed sHHP. Addition of age and applying a grading-system resulted in the HHPA, which appeared as the best prognostic model in the presented study. It represents a simple and objective option for early prognosis assessment of aSAH patients upon admission. Predictive performance remained strong in an external validation cohort suggesting adequate generalizability and low inter-rater variability.

## Supplementary information

Supplementary information.
