# Supplementary material for: Initial pupil status is a strong predictor for in-hospital mortality after aneurysmal subarachnoid hemorrhage
Source: Sci Rep. 2020 Mar 16;10:4764. doi: 10.1038/s41598-020-61513-1 (PMC7076009; doi:10.1038/s41598-020-61513-1)
Supplement: Supplementary file 1 — Supplementary information. [file 41598_2020_61513_MOESM1_ESM.pdf]

Supplementary information

-

Initial pupil status is a strong predictor for in-hospital mortality after aneurysmal subarachnoid hemorrhage

Marius M. Mader, MD[1], Andras Piffko, MD[1], Nora Dengler, MD[2], Franz L. Ricklefs, MD[1], Lasse, Dührsen, MD[1], Nils O. Schmidt, MD[1], Jan Regelsberger, MD[1], Manfred Westphal, MD[1], Stefan Wolf, MD[2], Patrick Czorlich, MD[1]

[1]*Department of Neurosurgery, University Medical Centre Hamburg-Eppendorf, Martinistraße 52, 20246 Hamburg, Germany*

[2]*Department of Neurosurgery, Charité-Universitätsmedizin Berlin, Charitéplatz 1, 10117 Berlin, Germany*

Corresponding author: Marius Marc-Daniel Mader, MD
Department of Neurosurgery
University Medical Center Hamburg-Eppendorf
Martinistraße 52
20246 Hamburg, Germany
Tel.: +49 (0) 40 7410 – 53750
Email: mader@uke.de

Supplementary figure 1

The pie charts show the distribution of pupil related scores (HHPA and sHHP) in relation to the grading of established scores (H&H and WFNS) of the derivation cohort. This is demonstrated for the whole cohort as well as for in-hospital mortality subgroups.

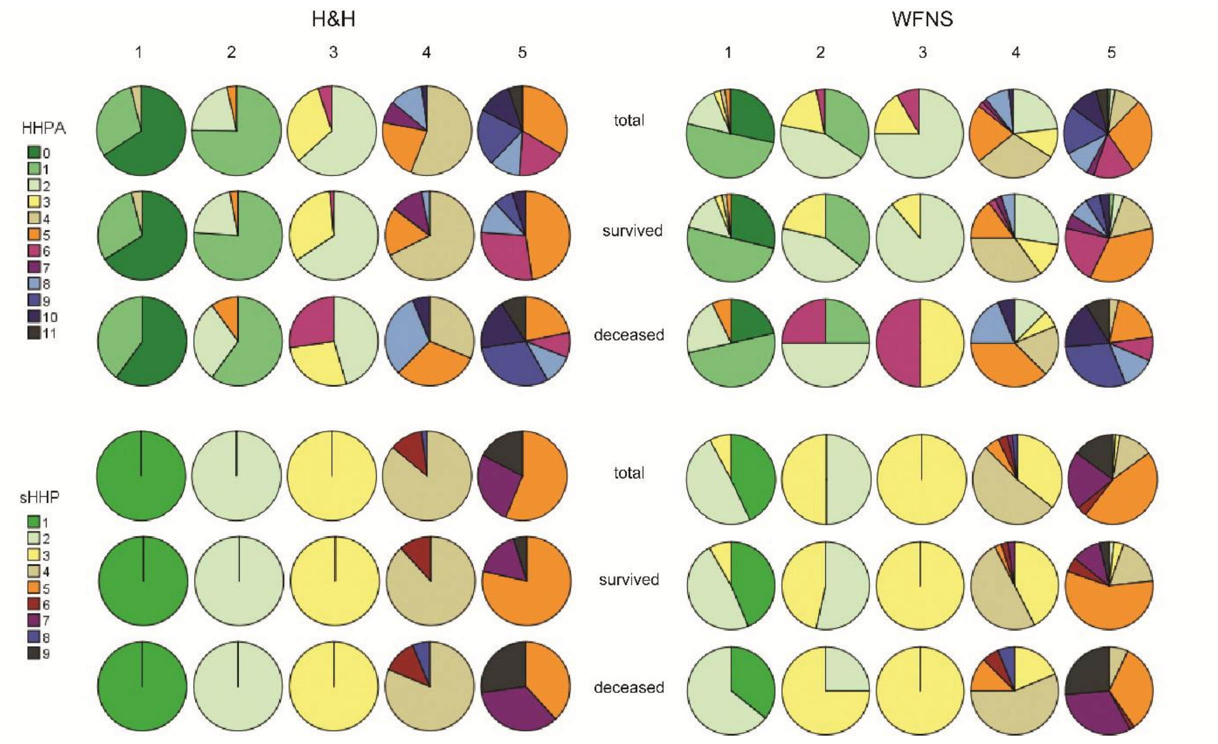
H&H
WFNS
1
2
3
4
5
1
2
3
4
5
HHPA
0
1
2
3
4
5
6
7
8
9
10
11
total
survived
deceased
sHHP
1
2
3
4
5
6
7
8
9
total
survived
deceased

Supplementary figure 2

The scatter plots demonstrate the time from bleed to a withdrawal of care decision (n = 40) in relation to HHPA, sHHP and GCS-P. A linear regression line is shown.

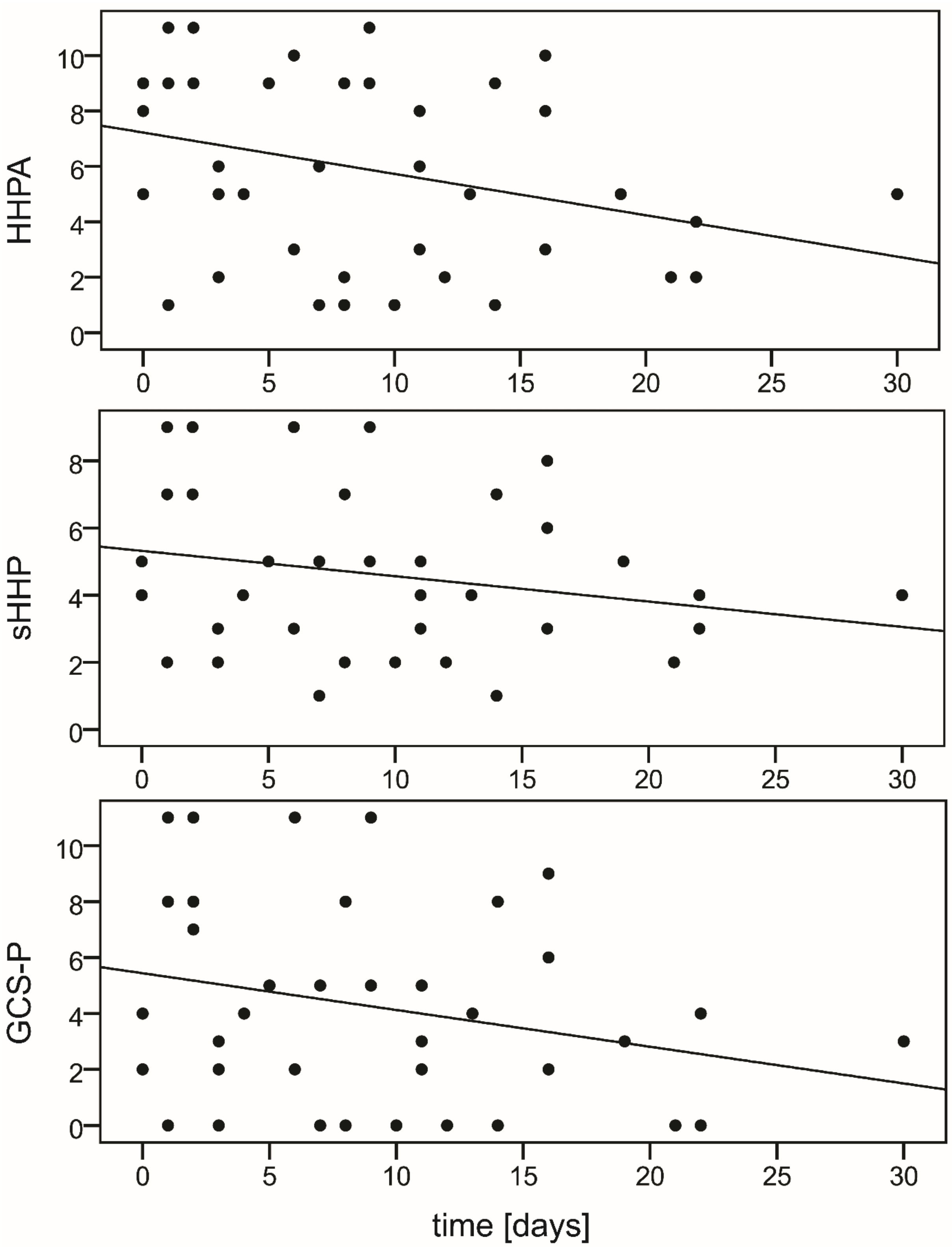

HHPA
sHHP
GCS-P
0
2
4
6
8
10
0
5
10
15
20
25
30
time [days]
